# Secular trends in adiposity in Norwegian 9-year-olds from 1999-2000 to 2005

**DOI:** 10.1186/1471-2458-9-389

**Published:** 2009-10-14

**Authors:** Elin Kolle, Jostein Steene-Johannessen, Ingar Holme, Lars B Andersen, Sigmund A Anderssen

**Affiliations:** 1 Department of Sports Medicine, Norwegian School of Sport Sciences, Oslo, Norway; 2 Research in Childhood Health, University of Southern Denmark, Odense, Denmark

## Abstract

**Background:**

Due to the negative health consequences of childhood obesity monitoring trends in body mass and adiposity is essential. The purpose of this study was to describe secular trends in the prevalence of overweight and obesity among 9-year-old children, and to study changes in adiposity and fat distribution by investigating changes in waist circumference (WC) and skinfold thicknesses.

**Methods:**

A total of 859 9-year-olds were included in two cross-sectional studies conducted in 1999-2000 and 2005. Measurements of body mass index (BMI; in kg/m^2^), WC and skinfold thicknesses were taken by trained investigators. The International Obesity Task Force cut-offs were used to define overweight and obese subjects.

**Results:**

The overall prevalence of overweight (including obesity) did not change over the five year period. However, a shift may have occurred as the prevalence of overweight (including obesity) increased by 6.4% in girls and 5.5% in boys over the five year period. In both study periods, logistic regression analyses revealed that children of non-Western origin had 2 times higher odds of being overweight/obese than those of Western origin. However, neither the children of Western origin nor the children of non-Western origin showed a significant increase in the prevalence of overweight over the five-year period. No changes were observed for mean BMI, while a significant increase in WC was reported for both girls and boys, and an increase in all skinfold measurements was observed in girls only. Shifts in percentile distribution were observed for BMI, WC and sum of 4 skinfold thickness, however, the shift appeared to be faster in the upper end of the population distribution (p < 0.001 for interactions).

**Conclusion:**

From 1999-2000 to 2005, there have been increases in 9-year-olds measures of adiposity even though the BMI did not change. The results indicate the need of a large-scale monitoring of adiposity, in addition to BMI, in children.

## Background

The prevalence of childhood overweight and obesity has increased over the past decades [1]. In the United States, 35% of school-aged children are currently classified as overweight or obese [2], while the corresponding numbers are 23% in Australian 2-16-year-olds [3], between 27 and 36% in 7-11-years-olds in Mediterranean countries [4], 20% in 4-18-year-olds in the UK [5] and 15-20% in 8-14-year-olds in the Scandinavian countries [6,7]. These findings are based on body mass index (BMI), and gender- and age-adjusted cut-offs developed by the International Obesity Task Force (IOTF) [8].

Although BMI is widely used as a surrogate measure of adiposity, it is a measure of excess weight relative to height, rather than excess body fat [9]. Furthermore, BMI fails to capture how body fat is distributed. Obesity is not a homogeneous condition, and several studies in adults have suggested that abdominal obesity is a better predictor of chronic diseases such as type 2 diabetes, hypertension and dyslipidemias, than overall adiposity assessed using BMI [10,11]. In children and adolescents, adverse concentrations of lipids and insulin have been shown to correlate well with abdominal obesity [12].

Children can experience both physical and psychological adverse effect of overweight and obesity. Furthermore, weight in childhood predicts weight in adulthood, and being overweight as an adult comes with a whole set of consequences of its own. It is therefore essential to monitor trends in the prevalence of overweight and obesity among children. However, fatness can change without changes in BMI and it is possible that children may be getting fatter at the expense of lean tissue, even if weight does not change [13]. Therefore, monitoring trends in BMI alone may not identify changes in body composition.

An examination of secular trends in adiposity in Norwegian children adds valuable comparative data to the evidence of trends in BMI. The study is also novel by including Tanner stage data. The aim of the current paper is to describe secular trends in the prevalence of overweight and obesity using IOTF cut-off values among 9-year-old children, and to study trends in adiposity and fat distribution by investigating changes in the children's waist circumference (WC) and skinfold thicknesses.

## Methods

### Participants

Participants were grade 4 girls and boys, living Oslo, Norway. Data were collected in two cross-sectional studies: the European Youth Heart Study, carried out in 1999-2000 and the Physical Activity among Norwegian Children Study, carried out in 2005. In 1999, all elementary schools in Oslo were stratified according to the socioeconomic character of their local area. A proportional sample of schools was randomly selected from each of three socioeconomic strata (low, middle and high) based on the number of students attending each school. In 1999-2000, children were recruited from the exact same nine elementary schools in Oslo, and the same schools were included in 2005. In 1999-2000, 410 of 578 invited children participated, giving a participation rate of 70.9%. In 2005, 449 of 491 invited children chose to participate, giving a participation rate of 91.4%. We obtained written, informed consent from the child's parent or legal guardian after they were given, in writing, a full explanation of the aims of the study and its possible hazards, discomfort, and inconvenience. The Regional Committee for Medical Research Ethics and Norwegian Social Science Data Services approved the study.

### Anthropometric measures

Children had a physical examination at their school. Trained investigators took all anthropometric measures, and an identical study protocol was used in both studies. Weight and height were measured in light clothing and without shoes. Weight was measured to the nearest 0.1 kg with a digital Seca 770 scale (SECA GmbH, Hamburg, Germany). Height was measured to the nearest 0.1 cm, using wall mounted tapes, with the child standing upright against the wall. BMI was calculated as weight (kg) divided by the height squared (m^2^).

Waist circumference was measured with a metal anthropometric tape midway between the lower rib and the iliac crest at the end of a normal expiration.

Four skinfold thickness measurements (triceps, biceps, subscapular and suprailiac) were taken using a Harpenden skinfold caliper (John Bull; British Indicators Ltd., West Sussex, England) on the left side of the body according to the criteria described by Lohman et al [14]. Duplicate measurements were taken for each skinfold, with a third measure taken if the difference between the two measurements differed by ≥ 2 mm, and the two closest measurements were averaged. No data on reliability and accuracy of anthropometric measures were reported in 1999-2000. In 2005, the intra-class (within-observer) correlation coefficients were ≥ 0.95 for skinfold measurements and 0.93 for WC, while the inter-class (between observers) correlation coefficients were ≥ 0.84 for skinfold measurements and 0.94 for WC. There was no systematic difference between first and second measurement in waist circumference or any of the skinfold measures. The difference in sum of 4 skinfolds was less than 1% (non-significant). We assessed the children's sexual maturity, using Tanner's 5-stage scale for breast development in girls and pubic hair in boys [15]. From the 859 subjects who participated, 788 subjects had complete anthropometric and sexual maturity data and were included in the analyses. Reasons for exclusion were missing BMI (n = 1), WC (n = 17), skinfold measures (n = 5), age (n = 1), sexual maturity (n = 37) or lacking all anthropometric measures (n = 10). There were no significant differences in BMI, WC, skinfold thickness or sexual maturity between the participants whose data were included or excluded (N = 71) due to missing data.

### Socio-demographic measures

The classification of socioeconomic status (SES) was based on the economic profile of the catchment area of the schools the children attended. Based on the average gross income per inhabitant aged between 30 and 66 years liable to pay taxes within the different school catchment areas, the local authorities calculated the percentage inhabitants who had a gross income considered as high (in 1997 defined as >50,000 US dollars). This calculation was used to divide the catchment areas into three gross income groups: low-, middle-, and high-SES areas. From these three subgroups we included four schools from low-SES areas, two schools from middle-SES areas and three schools from high-SES areas. The clustered SES code was assigned to each child within the dataset. In 1999-2000, 35% of the invited children from low-SES groups declined to participate in the study, whereas 23% and 21% of the invited children from middle- and high-SES groups, respectively, declined to participate. In 2005, 11%, 10% and 5% of the invited children from low-, middle- and high-SES groups, respectively, declined to participate in the study.

In 1999-2000, 84% of the participants were of western origin (Caucasian), while the corresponding number was 77% in 2005. The non-Western origin sample included black-African, black-other, Pakistani, Vietnamese, Chinese, Indian, Arab and Other individuals.

### Statistical analysis

All values are presented as mean (SD) unless otherwise stated. Children were classified as overweight or obese based on age- and gender-specific BMI cut-off points for children developed by the IOTF [8]. Four age intervals, 8.75-9.24, 9.25-9.74, 9.75-10.24 and 10.25-10.74 years were used to correspond to ages 9.0, 9.5, 10.0 and 10.5 years, respectively, by IOTF definition. As the prevalence of obesity was fairly low, the data from children classified as overweight or obese were merged for further analyses. The annualized change in the prevalence of overweight and obesity was calculated as ([prevalence at 2005 - prevalence at 1999-2000]/5).

Logistic regression was applied to study change in overweight (including obesity). The dependent variable in this model was the binary status of overweight (including obesity) and the study period was the main predictor, with the 1999-2000 study period as the reference category. The analyses were adjusted for school and sexual maturity, and the results are presented both as crude odds ratios (ORs) and as adjusted ORs with 95% confidence intervals (CI). Changes in anthropometric variables between study periods were tested by using general linear models. The anthropometric variable was defined as the dependent variable, study period was the fixed factor, and the analysis was adjusted for age, sexual maturity and school. The study sample was split by sex due to interactions between sex and study period.

Analysis of covariance (ANCOVA) was used to study the associations between SES and the anthropometric measures (BMI, WC and sum of 4 skinfold thicknesses) between the two study periods. The patterns of associations were compared among different SES groups by testing interaction terms (study period*SES). As SES can be confounded by ethnicity, all analyses are adjusted for ethnicity as well as age and sexual maturity.

Standardized change was assessed by investigating changes in Z-scores for BMI, WC and sum of 4 skinfold thicknesses ([mean 2005 - mean 1999-2000]/SD 1999-2000). Shift in BMI, distribution was assessed by using general linear model. BMI was defined as the dependent variable in the model, and study period and deciles of BMI were the main predictors. Shift in BMI distribution was investigated by testing interaction terms (study period*decile of BMI). The same procedure was performed to test for shift in WC and sum of 4 skinfold thicknesses. Statistical analyses were performed using the Statistical Package for the Social Sciences (SPSS, version 15.0).

## Results

Table 1 displays the characteristics of the study participants. The participants in 2005 were significantly older, taller, heavier, and had higher WC and sum of 4 skinfold thicknesses than the children in 1999-2000 (p < 0.01). Since the participants were significantly older in 2005, all analyses were adjusted for age. In both studies, 83% of the participants were prepubertal (Tanner stage I), and the rest were early pubertal (Tanner stage 2).

**Table 1:** Characteristics of the participants.

Participant Characteristic	1999-2000 (n = 348)	2005 (n = 440)
Age (years)	9.7 (0.3)	9.9 (0.3)
No (%) girls	174 (50)	193 (44)
Height (cm)	139.1 (6.2)	140.7 (6.4)
Weight (kg)	33.3 (5.8)	34.7 (6.7)
BMI (kg/m^2^)	17.1 (2.2)	17.4 (2.6)
Median (IQR)	16.7 (15.5 - 18.2)	16.7 (15.6 - 18.6)
Overweight (No, %)^1^	41 (11.8)	68 (15.5)
Obese (No, %)^1^	3 (0.9)	13 (3.0)
Waist circumference (cm)	60.5 (5.7)	64.7 (7.5)
Sum of 4 skinfolds (mm)	36.3 (17.1)	43.0 (24.6)
Socioeconomic status (No, (%)		
Low	135 (38.8)	157 (35.7)
Middle	75 (21.6)	93 (21.1)
High	138 (39.7)	190 (43.2)
Ethnic background (No, %)^2^		
Western origin	280 (84.3)	304 (76.8)
Non-western origin	52 (15.7)	92 (23.2)

Values are mean (SDs) unless otherwise stated.

BMI, body mass index

^1^Defined on basis of BMI according to IOTF criteria

^2^Percentage is for number with complete data on this variable - 16 participants in 1999-2000 and 44 participants in 2005 had missing data on this variable

In 1999-2000, 12.6% of girls and 10.9% of boys were classified as overweight, while an additional 1.1% of girls and 0.6% of boys were obese. By 2005, 15.5% of girls and 15.4% of boys were classified as overweight, while an additional 4.7% of girls and 1.6% of boys were classified as obese. Among girls, the prevalence of overweight and obesity increased by a total of 6.4% over the five year period, whereas the corresponding increase among boys was 5.5%. The increase was, however, not significant (p = 0.11 and 0.12 in girls and boys, respectively). The changes in overweight (including obesity) are presented in Table 2. In both study periods, logistic regression analyses revealed that children of non-Western origin had higher odds of being overweight than those of Western origin (1999-2000: OR = 2.3, 95% CI: 1.04, 5.00; 2005: OR = 2.0, 95% CI 1.1, 3.59). However, neither the children of Western origin nor the children of non-Western origin showed a significant increase in the prevalence of overweight over the five-year period.

**Table 2:** Crude prevalence and odds ratios (ORs) for prevalence of overweight (including obese) children.

	Number (n) and prevalence (%) of overweight and obese children						
				
	1999-2000		2005				
				
	N	%	N	%	Annualized change (%)	OR* (95% CI)	Adjusted OR*† (95% CI)
Girls	24	13.8	39	20.2	1.28	1.58 (0.91, 2.76)	1.58 (0.83, 2.98)
Boys	20	11.5	42	17.0	1.1	1.58 (0.89, 2.80)	1.43 (0.80, 2.56)
							
Western origin	30	10.7	45	14.8	0.82	1.45 (0.88, 2.37)	1.35 (0.81, 2.26)
Non-Western origin	13	25.0	28	30.4	1.08	1.31 (0.61, 2.83)	1.45 (0.61, 3.46)

*The year 1999-2000 was used as the reference category.

‡Analyses are adjusted for school and sexual maturity

Table 3 shows the mean values for BMI, WC and skinfold thicknesses. Between the periods of 1999-2000 and 2005, no change was seen for mean BMI, however, the age-adjusted mean WC increased from 60.1 cm to 65.8 cm among girls (p < 0.001), and from 61.5 cm to 63.4 cm among boys (p < 0.001). In girls, significant increase was observed for all skinfold measurements. The differences ranged from 2.3 to 3.0 mm at the four sites, while the sum of 4 skinfold thicknesses increased by 10.5 mm which translates into a 25.7% difference (95% CI: 21.2, 30.2). In boys, increase in triceps and supra iliac skinfold thicknesses were borderline significant (p = 0.05), while no changes were observed for the other skinfold measurements.

**Table 3:** Mean* (SE) anthropometric data among the boys and girls in the two study samples, by sex.

	1999-2000	2005	Mean difference (95% CI)	P Value
**Girls**	n = 174	n = 193		
BMI (kg/m^2^)	17.3 (0.2)	17.7 (0.2)	0.4 (-0.1, 0.9)	0.08
Waist circumference (cm)	60.1 (0.5)	65.8 (0.5)	5.7 (4.4, 7.0)	< 0.001
Triceps SF (mm)	13.1 (0.4)	15.4 (0.3)	2.3 (1.3, 3.3)	< 0.001
Biceps SF(mm)	8.9 (0.3)	11.2 (0.3)	2.3 (1.4, 3.2)	< 0.001
Subscapula SF (mm)	9.5 (0.5)	12.5 (0.5)	3.0 (1.7, 4.3)	< 0.001
Suprailiac SF (mm)	9.1 (0.5)	11.8 (0.4)	2.7 (1.4, 4.0)	< 0.001
Sum 4 SF (mm)	40.5 (1.5)	50.9 (1.4)	10.5 (6.4, 14.6)	< 0.001
**Boys**	n = 174	n = 247		
BMI (kg/m^2^)	17.1 (0.2)	17.2 (0.1)	0.1 (-0.5, 0.5)	0.92
Waist circumference (cm)	61.5 (0.5)	63.4 (0.4)	1.9 (0.6, 3.2)	0.005
Triceps SF (mm)	10.8 (0.4)	11.7 (0.3)	1.1 (0.1, 2.1)	0.05
Biceps SF(mm)	7.3 (0.3)	7.9 (0.3)	0.6 (-0.3, 1.5)	0.19
Subscapula SF (mm)	7.8 (0.4)	8.1 (0.3)	0.3 (-0.8, 1.4)	0.58
Suprailiac SF (mm)	7.1 (0.5)	8.5 (0.4)	1.4 (0.1, 2.7)	0.05
Sum 4 SF (mm)	33.0 (1.5)	36.2 (1.3)	3.2 (-0.7, 7.1)	0.12

*Values are adjusted for age, sexual maturity and school. CI, Confidence Interval; BMI, Body Mass Index; SF, Skinfold.

In 1999-2000, there was no association between SES and BMI, WC and sum of 4 skinfold thicknesses neither in girls nor in boys. In 2005, girls from high-SES groups had significantly lower BMI (p = 0.012), WC (p = 0.001) and sum of 4 skinfold thicknesses (p = 0.002) than girls from low-SES groups. In girls, a borderline significant interaction was found between study period and SES for WC (p = 0.057), and a significant interaction was found between SES and study period for sum of 4 skinfold thicknesses (p = 0.037). Girls in all three SES groups had an increase in WC and sum of 4 skinfold thicknesses, but the increase was larger in girls from low-SES groups than in girls from high-SES groups.

**Figure 1:**
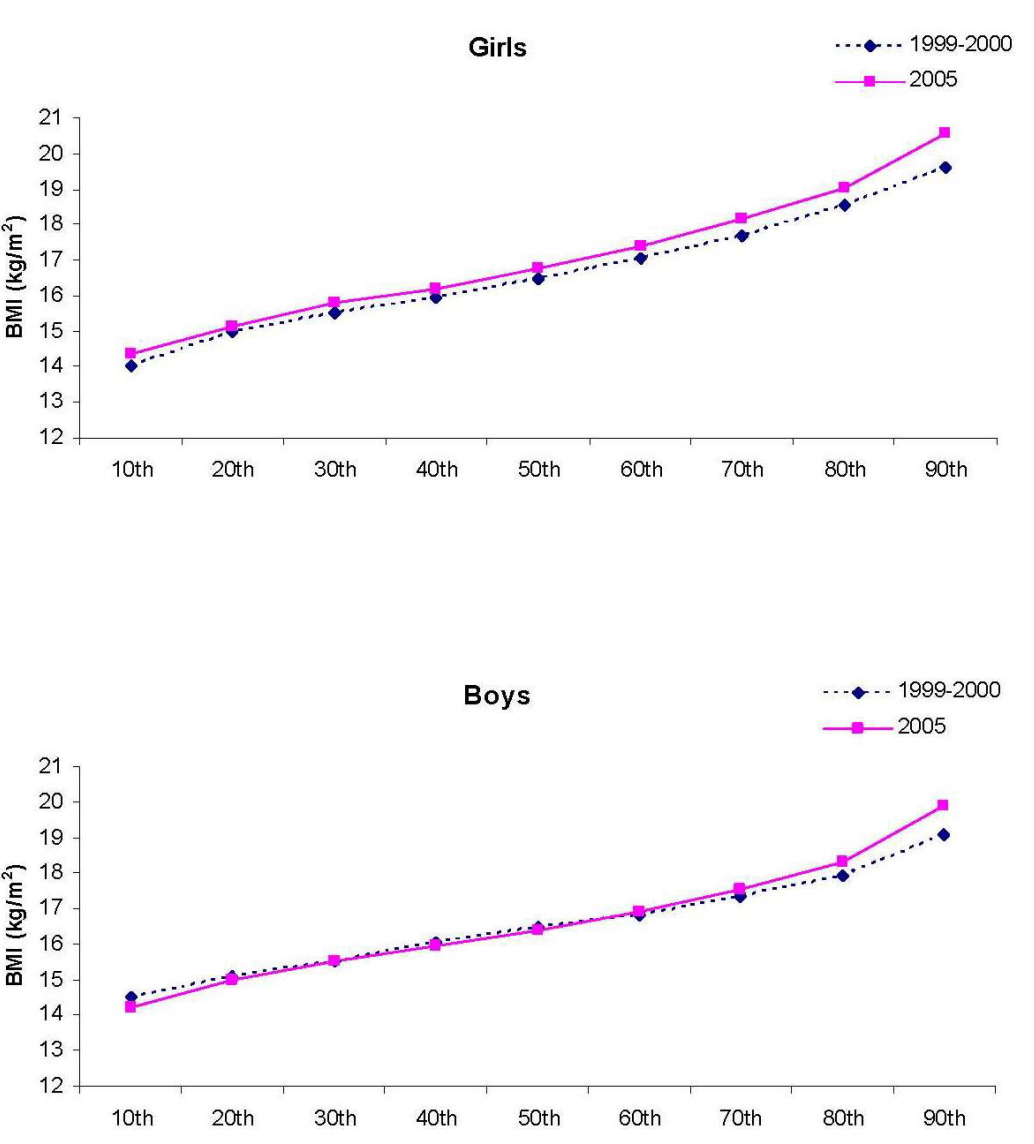
**The 10th to the 90th percentiles in BMI in girls and boys in 1999-2000 and 2005**.

**Figure 2:**
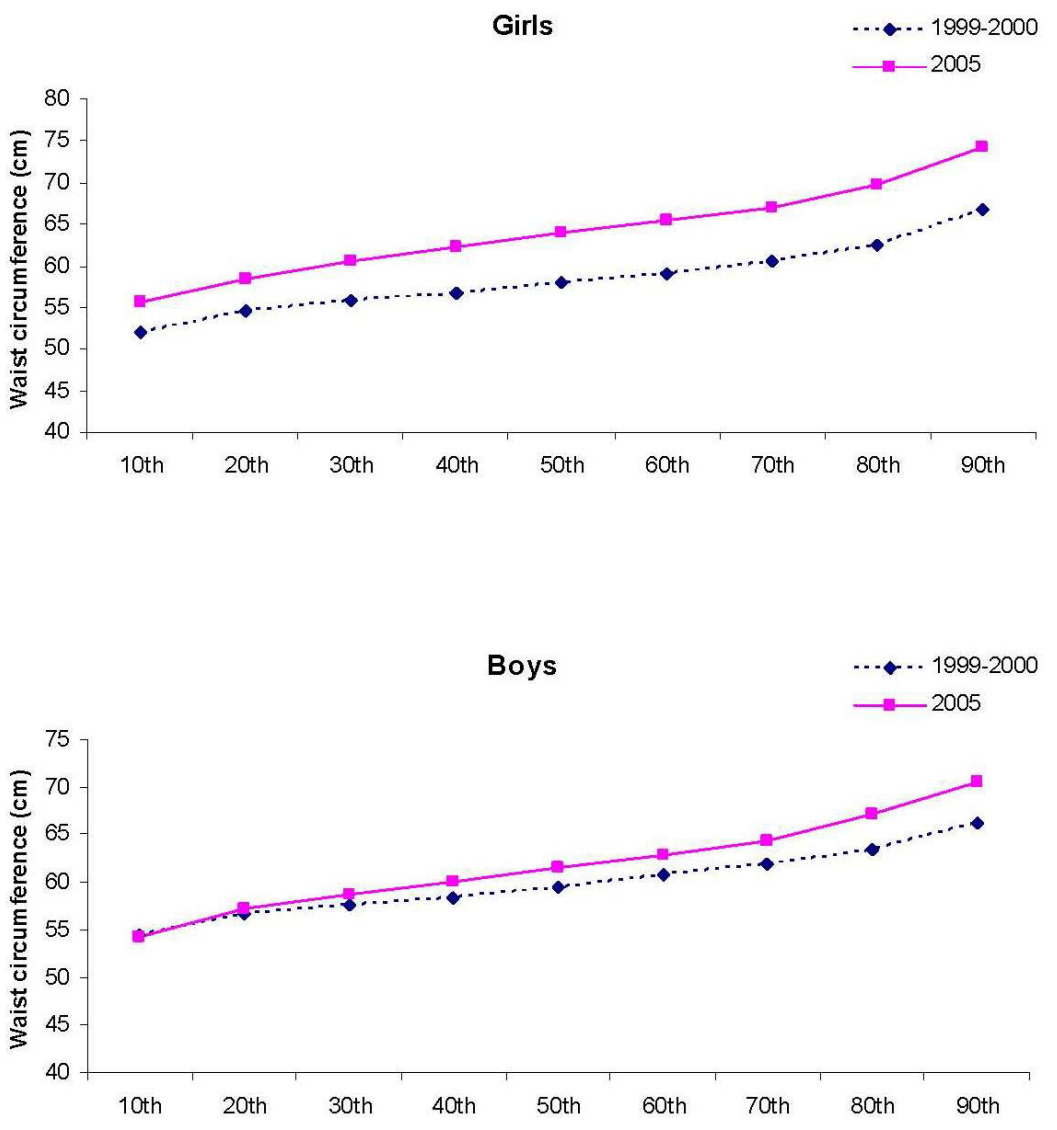
**The 10th to the 90th percentiles in waist circumference in girls and boys in 1999-2000 and 2005**.

**Figure 3:**
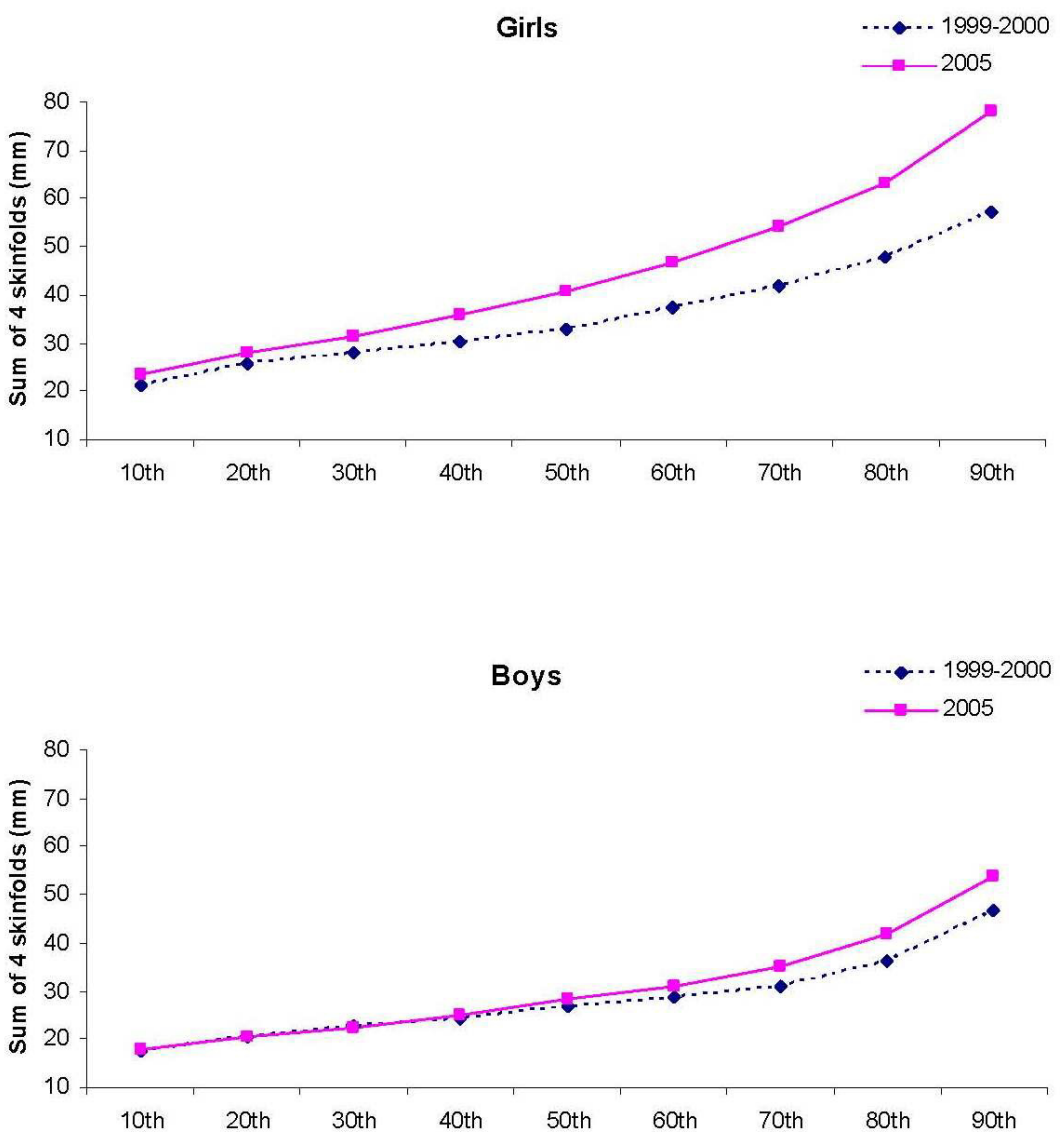
**The 10th to the 90th percentiles in sum of 4 skinfolds in girls and boys in 1999-2000 and 2005**.

Among boys, the pattern of shift (increase) of BMI appeared in the upper percentiles, whereas the increase of WC and sum of 4 skinfold thickness appeared approximately in the 40th to 50th percentile (Figure 1, 2, 3). In contrast, shifts in the girls' values were observed in all percentiles, but BMI, WC, and sum of 4 skinfold thicknesses appeared to increase faster in the upper end of the population distribution. There appeared to be a shift to higher values at the upper end of the population distribution (p < 0.001 for interaction between study period and deciles of BMI, WC and sum of four skinfolds). Among girls, the absolute change in 1999-2000 SD-units (change in Z-score) was 0.21 for BMI, 1.0 for WC and 0.61 for sum of 4 skinfold thicknesses. The corresponding values for boys were 0.09, 0.47 and 0.27.

## Discussion

In the current study, no statistically significant change in the prevalence of overweight (including obesity) was found in 9-year-old girls and boys between 1999-2000 and 2005. However, a shift may have occurred as the prevalence of overweight (including obesity) increased by 6.4% in girls and 5.5% in boys over the 5 year period. The magnitude of the rise is in line with reports elsewhere [1]. A recent study from the US [16] reported no increase in childhood obesity between 2003-04 and 2005-06, however, results are inconsistent. A Swedish study [7] reported a decrease in the prevalence of childhood overweight (including obesity) among girls from 2000-01 to 2004-05, while a study from Cyprus reported an increase in obesity among 11-year-olds from 1997-98 to 2002-03 [17]. The differences might be explained by different time periods and different age groups of the children included in the studies. Also, some studies included national representative samples, while others used small, non-representative samples. As these factors varied across studies comparability of findings is difficult.

In both study periods, race/ethnicity was associated with prevalence of overweight and obesity as has been reported in other countries. In the US, higher prevalence of overweight has been reported among children who are non-Hispanic black or Mexican American than among children who are non-Hispanic white [16]. In the UK, British Afro-Caribbean and Pakistani girls have increased risk of being obese, while Indian and Pakistani boys have an increased risk of being overweight than the general population [18]. Despite this, we did not observe an increase in the prevalence of overweight (including obesity) in either children of western or non-western origin over the five years period. This indicates that the prevalence of overweight is not rising faster among minority groups than in the general population. Similar trends have recently been reported in the US [16].

Our results revealed that where there was no change in mean BMI, there was a change in body shape and body composition. In particular, girls increased their WC by 5.7 cm in five years; an increase of 1.1 cm annually. In addition, the increased skinfold thicknesses observed in girls indicates increased adiposity, which are all factors associated with higher disease risk [19]. Our results are in accordance with several cross-sectional studies showing increases in WC [20,21] and skinfold thickness [22,23] that are greater than increases in BMI. Similar results have also been seen in a longitudinal study that reported a steeper increase in waist circumference compared to BMI between 7-8 to 12-13 years [24]. As was the case in our study, the increase was greater in girls than in boys in all previous studies. Different reasons for this sex difference have been suggested, such as sexually diverse changes in body composition that occur during puberty, or decreased habitual physical activity among girls. However, we found no evidence for an increase in the prevalence of early puberty, as an equal number of girls were categorized in Tanner stage 2 at both measurement points. Further, an increase in objectively assessed physical activity was seen in both girls and boys in our study population[25]. From 1999-2000 to 2005, the children increased their mean physical activity level assessed by accelerometers. In the present study, an interaction was reported between SES and WC and skinfold thicknesses in girls. A similar pattern was observed with regards to physical activity. Between the study periods, girls from high-SES groups had a significant increase in their mean physical activity that was not observed among girls from low-SES groups. At the same time the latter group had a higher increase in WC and skinfold thickness compared to the girls from the low-SES groups.

In both girls and boys, the distribution of BMI, WC and sum of 4 skinfold thicknesses appeared to increase faster in the upper end of the distribution. The mentioned variables appeared to change with greater amounts among girls compared to boys. Increase in the upper BMI-percentiles have also been reported in previous studies [26-30]. Bjørnelv et al [27] reported decreases in the lowest BMI percentiles that was not observed in the present study. The increase of especially WC and skinfold thicknesses indicate that the heavier children became heavier, and the fat children became fatter. The increase in the upper end of the population may partly be caused by the higher participation rate in 2005 (91.4%) than in 1999-2000 (70.9%). We have no data on the children who did not participate in the study, but as the aim was to measure adiposity, it is possible that the heaviest children chose not to participate. If this is true, the changes in WC and skinfold thicknesses may be somewhat overestimated.

It is not likely that the changes in anthropometric variables are due to methodological differences between studies. In both studies the sites of the measurements were defined identically, and the investigators in 2005 were trained thoroughly by the project leader in 1999-2000 with the aim of collecting comparable anthropometric data at all examinations. Also, with a high intra-observer and inter-observer correlation, it is likely that the increases in WC and skinfold thickness are genuine.

Our findings have some important public health implications. Over a few years, body composition of 9-year-olds has changed with increased skinfold thickness and central obesity. As mean BMI did not change, this indicates a decrease in muscle mass which is of concern because resting metabolic rate is highly correlated with body weight in general and with muscle mass in particular. Hence, increased fat tissue and decreased muscle mass leads to decreased resting metabolic rate, decreased energy expenditure and the consequence is further increase in body weight. This highlights and emphasizes the important role of physical activity in both preventing and treating overweight and obesity in children. Furthermore, our results support the suggestion from Dollman and Olds [20] that the reported increase in the prevalence of obesity, based on BMI, may be an underestimate of the true extent of the problem. The results from the present paper indicate that there is a need for large-scale monitoring of fatness per se in children. Skinfold measurements have been used widely to assess obesity and are considered good indicators since they directly measure a layer of subcutaneous fat. The method is also simpler to use than laboratory techniques and the costs are minimal, however, it is open to random and systematic errors [13]. Waist circumference is relatively easy to measure, reliable and low cost. Measures of waist circumference should, in addition to measures of height and weight, be standardized, and routinely incorporated in clinical and epidemiological settings, as well as in school health examinations.

A strength of this study is that several objective measures of body mass and body composition were included. Further, the participation rates in both studies were high and the samples described here are representative of the population of that age and time period in Oslo, Norway. A limitation of the study is the inclusion of one single age-group. It would be useful to know if the observations reported here are true for children of all age-groups. Also, all children in the present paper are classified as normal weight, overweight or obese. It is important to mention that the normal weight category is likely to include underweight children and this is a limitation of the study.

## Conclusion

In conclusion, we found that in Norwegian children aged 9-years-old there has been no change in the prevalence of overweight (including obesity) over a five year period. However, a shift may have occurred as the prevalence of overweight (including obesity) increased by 6.4% in girls and 5.5% in boys over the 5 year period. Rapid increases in WC and skinfold thickness were seen, indicating increased adiposity. Our results emphasize the need to monitor trends in waist circumference and skinfold thickness, in addition to BMI, to be able to identify changes in body composition in children.

## List of abbreviations

BMI: body mass index; CI: confidence interval; IOTF: International Obesity Task Force; SF: skin fold; WC: waist circumference.

## Competing interests

The authors declare that they have no competing interests.

## Authors' contributions

EK and JSJ were responsible for conception of the study, study design and coordinated and participated in the data collection. IH, LBA and SAA were involved with conception of the study and study design. EK analyzed the data and drafted the manuscript. All authors were involved with data interpretation, critical revisions of the paper and provided approval for its publication.

## Pre-publication history

The pre-publication history for this paper can be accessed here:

http://www.biomedcentral.com/1471-2458/9/389/prepub

## References

[B1] Wang Y Lobstein T Worldwide trends in childhood overweight and obesity Int J Pediatr Obes 2006 9 11 25 10.1080/17477160600586747 17902211

[B2] Lobstein T Jackson-Leach R Child overweight and obesity in the USA: prevalence rates according to IOTF definitions Int J Pediatr Obes 2007 9 62 64 10.1080/17477160601103948 17763012

[B3] Australian Government - Department of Health and Aging. 2007 Australian National Children's Nutrition and Physical Activity Survey - Main Findings. 1-44. 2008. Australian Government - Department of Health and Aging; Australian Food and Grocery Council; Australian Government - Department of Agriculture, Fisheries and Forestry Ref Type: Report

[B4] Lobstein T Frelut ML Prevalence of overweight among children in Europe Obes Rev 2003 9 195 200 10.1046/j.1467-789X.2003.00116.x 14649370

[B5] Jebb SA Rennie KL Cole TJ Prevalence of overweight and obesity among young people in Great Britain Public Health Nutr 2004 9 461 465 10.1079/PHN2003539 15153277

[B6] Andersen LF Lillegaard IT Overby N Lytle L Klepp KI Johansson L Overweight and obesity among Norwegian schoolchildren: changes from 1993 to 2000 Scand J Public Health 2005 9 99 106 10.1080/140349404100410019172 15823970

[B7] Sjoberg A Lissner L bertsson-Wikland K Marild S Recent anthropometric trends among Swedish school children: evidence for decreasing prevalence of overweight in girls Acta Paediatr 2008 9 118 123 10.1111/j.1651-2227.2007.00613.x 18201312

[B8] Cole TJ Bellizzi MC Flegal KM Dietz WH Establishing a standard definition for child overweight and obesity worldwide: international survey BMJ 2000 9 1240 1243 10.1136/bmj.320.7244.1240 10797032 PMC27365

[B9] Horlick M Body mass index in childhood--measuring a moving target J Clin Endocrinol Metab 2001 9 4059 4060 10.1210/jc.86.9.4059 11549625

[B10] Yusuf S Hawken S Ounpuu S Bautista L Franzosi MG Commerford P Lang CC Rumboldt Z Onen CL Lisheng L Tanomsup S Wangai P Jr Razak F Sharma AM Anand SS INTERHEART Study Investigators Obesity and the risk of myocardial infarction in 27,000 participants from 52 countries: a case-control study Lancet 2005 9 1640 1649 10.1016/S0140-6736(05)67663-5 16271645

[B11] Pischon T Boeing H Hoffmann K Bergmann M Schulze MB Overvad K Schouw YT van der Spencer E Moons KG Tjønneland A Halkjaer J Jensen MK Stegger J Clavel-Chapelon F Boutron-Ruault MC Chajes V Linseisen J Kaaks R Trichopoulou A Trichopoulos D Bamia C Sieri S Palli D Tumino R Vineis P Panico S Peeters PH May AM Bueno-de-Mesquita HB van Duijnhoven FJ Hallmans G Weinehall L Manjer J Hedblad B Lund E Agudo A Arriola L Barricarte A Navarro C Martinez C Quirós JR Key T Bingham S Khaw KT Boffetta P Jenab M Ferrari P Riboli E General and abdominal adiposity and risk of death in Europe N Engl J Med 2008 9 2105 2120 10.1056/NEJMoa0801891 19005195

[B12] Freedman DS Serdula MK Srinivasan SR Berenson GS Relation of circumferences and skinfold thicknesses to lipid and insulin concentrations in children and adolescents: the Bogalusa Heart Study Am J Clin Nutr 1999 9 308 317 9989697 10.1093/ajcn/69.2.308

[B13] Livingstone B Epidemiology of childhood obesity in Europe Eur J Pediatr 2000 9 Suppl 1 S14 S34 10.1007/PL00014363 11011953

[B14] Lohman TG Roche AFM Martorell R Anthropometric standardization reference manual 1991 Champaign, IL: Human Kinetics

[B15] Tanner JM Growth at adolescence 1962 Oxford, United Kingdom: Blackwell

[B16] Ogden CL Carroll MD Flegal KM High body mass index for age among US children and adolescents, 2003-2006 JAMA 2008 9 2401 2405 10.1001/jama.299.20.2401 18505949

[B17] Savva SC Tornaritis MJ Chadjigeorgiou C Kourides YA Siamounki M Kafatos A Prevalence of overweight and obesity among 11-year-old children in Cyprus, 1997-2003 Int J Pediatr Obes 2008 9 186 192 10.1080/17477160701705451 18612872

[B18] Saxena S Ambler G Cole TJ Majeed A Ethnic group differences in overweight and obese children and young people in England: cross sectional survey Arch Dis Child 2004 9 30 36 14709498 PMC1755912

[B19] Lobstein T Baur L Uauy R Obesity in children and young people: a crisis in public health Obes Rev 2004 9 Suppl 1 4 85 10.1111/j.1467-789X.2004.00133.x 15096099

[B20] Dollman J Olds TS Secular changes in fatness and fat distribution in Australian children matched for body size Int J Pediatr Obes 2006 9 109 113 10.1080/17477160600684260 17907323

[B21] McCarthy HD Ellis SM Cole TJ Central overweight and obesity in British youth aged 11-16 years: cross sectional surveys of waist circumference BMJ 2003 9 624 10.1136/bmj.326.7390.624 12649234 PMC151972

[B22] Juliusson PB Roelants M Eide GE Hauspie R Waaler PE Bjerknes R Overweight and obesity in Norwegian children: secular trends in weight-for-height and skinfolds Acta Paediatr 2007 9 1333 1337 10.1111/j.1651-2227.2007.00421.x 17718787

[B23] Moreno LA Fleta J Sarria A Rodriguez G Gil C Bueno M Secular changes in body fat patterning in children and adolescents of Zaragoza (Spain), 1980-1995 Int J Obes Relat Metab Disord 2001 9 1656 1660 10.1038/sj.ijo.0801803 11753587

[B24] Garnett SP Cowell CT Baur LA Shrewsbury VA Chan A Crawford D Salmon J Campbell K Boulton TJ Increasing central adiposity: the Nepean longitudinal study of young people aged 7-8 to 12-13 y Int J Obes 2005 9 1353 1360 10.1038/sj.ijo.0803038 16077716

[B25] Kolle E Steene-Johannessen J Klasson-Heggebo L Andersen LB Anderssen SA A 5-yr Change in Norwegian 9-yr-Olds' Objectively Assessed Physical Activity Level Med Sci Sports Exerc 2009 9 1368 1373 10.1249/MSS.0b013e31819a5e65 19516165

[B26] Ekblom O Oddsson K Ekblom B Prevalence and regional differences in overweight in 2001 and trends in BMI distribution in Swedish children from 1987 to 2001 Scand J Public Health 2004 9 257 263 10.1080/1403494031009498 15370765

[B27] Bjornelv S Lydersen S Mykletun A Holmen TL Changes in BMI-distribution from 1966-69 to 1995-97 in adolescents. The Young-HUNT study, Norway BMC Public Health 2007 9 279 10.1186/1471-2458-7-279 17916233 PMC2082034

[B28] Dollman J Olds T Norton K Stuart D The evolution of fitness and fatness in 10-11-year-old Australian schoolchildren: Changes in distributional characteristics between 1985 and 1997 Pediatric Exercise Science 1999 9 108 121

[B29] Flegal KM Troiano RP Changes in the distribution of body mass index of adults and children in the US population Int J Obes Relat Metab Disord 2000 9 807 818 10.1038/sj.ijo.0801232 10918526

[B30] Troiano RP Flegal KM Overweight children and adolescents: description, epidemiology, and demographics Pediatrics 1998 9 497 504 12224656

